# Efficacy & safety of brolucizumab 6.0 mg versus 3.6 mg in diabetic macular edema

**DOI:** 10.1186/s40942-025-00628-x

**Published:** 2025-01-13

**Authors:** Sanjay Kumar Mishra, Pradeep Kumar, Amrita Joshi, Aman Saraf, Abhijeet Awasthi, Supriya Dhar, Khaleel M, Atul Kumar, Vipin Rana, Ravi D

**Affiliations:** 1 Army Hospital Research & Referral, Delhi Cantt, New Delhi, Delhi 110010 India; 2 Command Hospital Eastern Command, Kolkata, India

**Keywords:** Diabetic macular edema, DME, Brolucizumab, VEGF, India

## Abstract

**Background:**

Management of Diabetic Macular edema (DME) requires repeated injections. Therefore newer Anti-VEGFs like Brolucizumab with longer durability have been introduced. We compared two different dosages of Brolucizumab, 6.0 mg and 3.6 mg, for their safety & efficacy in treatment of DME, in treatment naïve patients over 52 weeks.

**Method:**

A prospective, pilot randomised controlled, single centre, double blinded, two arm comparative study was conducted between Dec 2022 to Apr 2024. The study recruited 82 patients of DME who were randomised into two groups of 41 patients each, one group to be treated with Brolucizumab 6.0 mg in 50 μL and the other to receive 3.6 mg in 30 μL. All patients received the first dose of Brolucizumab at 0 week and were then followed up at every 4 weeks for detailed ophthalmic and OCT macula examination. Those who met the pre-defined re-treatment criteria were re-injected with Brolucizumab, the dose being fixed for each group throughout the study. All patient receiving an injection were further followed up on Day 1, Day 7 and Day 28 to look for any adverse reactions. The efficacy parameters included change in best corrected visusal acuity (BCVA), contrast and central macular thickness (CMT) on Optical Coherence Tomography. The average number of injections recd in each group were also calculated.

**Results:**

The change in BCVA from baseline in 6.0 mg group was 0.54 LogMAR units and 3.6 mg group was 0.59 LogMAR units, which was not statistically significant. The reduction in CMT from baseline in 6.0 mg group was 133.2 µm (μ) and 3.6 mg group was 110.6 μ, which was not statistically significant. The improvement in contrast from baseline in 6.0 mg group was 0.74 and 3.6 mg group was 0.95, with p value of 0.0002. The re-injection interval was 14.21 weeks in 6.0 mg group and 15.56 weeks for 3.6 mg subgroup. The total number of adverse events in both groups were similar at 70 in 6.0 mg group and 47 in 3.6 mg group with only one grade 4 adverse event occurring in each group.

**Conclusion:**

The results of present study show that the safety and efficacy of both doses of Brolucizumab, i.e. 6.0 mg and 3.6 mg, for treating diabetic macular edema is similar.

*Trial registration* Study was registered with Clinical trials registry of India (CTRI ref no. CTRI/2023/06/054105), registered on 14 Nov 2022.

**Supplementary Information:**

The online version contains supplementary material available at 10.1186/s40942-025-00628-x.

## Background

Diabetic retinopathy (DR) has recently become the leading cause of irreversible blindness amongst adults in India with a major part of the visual loss being attributable to diabetic macular edema (DME) [1].

In the management of DME, strict diabetic control and optimization of systemic aggravating factors are crucial. Besides these three ophthalmic therapeutic modalities are available: first, laser therapy- including focal and grid laser [2], Second, corticosteroid therapy, involving intravitreal injections of triamcinolone acetonide and long-term dexamethasone implants [3], and third option is use of Anti- Vascular endothelial Growth Factor (VEGF) molecules.

The Anti-VEGF options available today include Bevacizumab, Ranibizumab, Aflibercept, Brolucizumab and Faricimab [4]. Out of these available options we need a molecule which is potent, efficacious, safe and cost effective. Ranibizumab had been the principal drug used for treatment of DME from 2010–2020 with robust evidence in the form of RESTORE trial followed by RISE and RIDE trial [5]. Aflibercept has gained wider acceptance due to its 8-weekly dosing and stronger drying effect. The VISTA & VIVID trials had given considerable evidence in support of using Aflibercept for DME [6].

Brolucizumab is a fragmented antibody molecule with a very small size of 27 kilodaltons [7]. Due to its smaller size the drug penetrates ocular tissues faster and more ranibizumab equivalent drug can be loaded into a single dosage of brolucizumab compared to other drugs. The KITE and KESTREL study provided ample evidence in support of safety and efficacy of brolucizumab in managing patients of diabetic macular edema [8]. The KESTREL study had 3 arms which included brolucizumab 3.0 mg, brolucizumab 6.0 mg and aflibercept 2.0 mg. A comparison of brolucizumab 3.0 mg subgroup with the brolucizumab 6.0 mg subgroup shows that the efficacy of the drug was almost equivalent in both the dosages.

The authors were interested in the unique drying capability and alleged longer duration of action of 12 weekly intervals with brolucizumab, a major advantage in reducing the number of hospital visits and the corresponding loss of men-hours and transportation and accommodation costs for the patient [7]. The authors wanted to compare a smaller dosage of brolucizumab compared to the recommended dosage of brolucizumab by its manufacturers. The basis of this thought process was the already proven track record of brolucizumab 3.0 mg in the KESTREL study. Further, a similar dose difference in the application of a particular Anti-VEGF drug between treatment of neovascular age related macular degeneration and diabetic macular edema already exists. Ranibizumab has been approved by The US FDA at the dosage of 0.3 mg in DME which is about 60% of the dosage recommended for treatment of neovascular age related macular degeneration (AMD) i.e. 0.5 mg [5]. The authors believe that a smaller dosage is still effective in managing DR as the principal targets are situated on the inner retina compared to the drug targets in neovascular AMD which are sub retinal or sub retinal pigment epithelium in nature. A deviation from the KITE and KESTREL study was where we wanted to see the real world effect of this drug with adoption of “pro re nata” regimen from the very beginning of the treatment rather than loading dosages followed by PRN treatment [8].

## Methods

### Purpose of the study

The purpose of our study was to analyse the changes in central macular thickness (CMT), contrast sensitivity and best corrected visual acuity (BCVA) after intravitreal Brolucizumab 6.0 mg versus 3.6 mg in treatment naive centre involving DME. The primary end point was the difference in central macular thickness by optical coherence tomography from baseline to 52 weeks. Secondary objectives were to assess the effect of intravitreal Brolucizumab on best corrected distance, and contrast sensitivity.

### Aim

To compare the changes in central macular thickness, visual acuity and contrast sensitivity after intravitreal Brolucizumab 6.0 mg versus 3.6 mg in treatment of naïve centre involving DME.

### Objectives

1. To compare the changes in CMT in treatment naïve centre involving DME eyes, after intravitreal Brolucizumab 6.0 mg versus 3.6 mg.
2. To compare the changes in best corrected visual acuity & contrast sensitivity in treatment naïve centre involving DME eyes, after intravitreal Brolucizumab 6.0 mg versus 3.6 mg.
3. To study the safety of Brolucizumab 6.0 mg versus 3.6 mg in DME

### Study design & approvals

The present study was a prospective, pilot randomised controlled, single centre, double blinded, two arm comparative study assessing the safety & efficacy of Brolucizumab 6.0 mg versus 3.6 mg in Diabetic Macular Edema (DME).

### Compliance

Clinical trials registry of India (https://ctri.nic.in/Clinicaltrials/login.php) (CTRI ref no. CTRI/2023/06/054105), registered on 14 Nov 2022. The study protocol was approved by the local Institutional Ethics Committee of Army Hospital Research & Referral, Delhi Cantt. (Approval number 144/22 dated 02 September 2022). The trial was conducted compliant with the principles of the Declaration of Helsinki. Prior to recruitment in the study, written informed consent was obtained from all the participants.

### Study population

During the period from Dec 2022 to Dec 2023, All diabetic patients presenting to this tertiary eye care institute during study period were screened for centre involving diabetic macular edema (CDME) with dilated direct and indirect ophthalmoscopy, biomicroscope examination of fundus with volk digital high magnification non-contact lens and OCT (Optical coherence tomopgraphy).

### Sample size calculation

Assuming Zα = 1.96 (95% CI), Z1-β = 1.28 (β = 10%, power = 90%), and.

Assuming a standard deviation of 3.6 letter per group and estimated effect size (Δ) 2.8 (taking reference from DA Vinci et al.)^36^

### Applying formula

$${\text{n}}\,{ = }\,{2}\left( {{\text{Z}}\alpha {\text{ + Z1 - }}\beta } \right) \, \left( {{2}\sigma } \right){ 2/}\Delta {2}$$

comes out to be 70, i.e. 35 patients in each arm. Accounting for 20% loss and attrition, 41 participants in each arm.

### Randomization methodology

Randomization was achieved using random sequence generators. Register was maintained by an independent observer to note the allocation of sequence numbers and the treatment group. The intravitreal injection was given by a masked physician.

Subgroups: Patients were randomly assigned to 2 subgroups in allocation arms 1:1 i.e. two groups with equal size, 41 in each arm (to offset patient loss during study), receiving intravitreal Brolucizumab 6.0 mg (Group 1) and 3.6 mg (Group 2) respectively.

### Inclusion criteria

1. Age more than 18 years with type 2 diabetes.
2. Centre involving diabetic macular edema.
3. BCVA better than 20/400 or LogMAR 1.3

### Exclusion criteria

1. Media opacity precluding OCT measurement at inception due to media opacities such as corneal opacity, dense cataracts, intravitreal haemorrhage etc. interfering with OCT acquisition.
2. Prior treated with Laser photocoagulation
3. Prior Anti VEGF administration or intravitreal steroids.
4. Decreased visual acuity due to causes other than diabetic macular edema such as a significant macular and optic nerve pathology, age-related macular degeneration, cataract, glaucoma, active or healed uveitis or other active ocular infection or inflammation.
5. Macular edema due to unknown cause/causes other than Diabetes mellitus.
6. Eyes with bad quality OCT Scan i.e. signal strength less than 6/10.
7. Further, the following systemic exclusion criteria were imposed: uncontrolled DM (HbA1c more than 10.0%), history of cerebro vascular accident or myocardial infarction within 3 months, renal failure requiring dialysis or renal transplant, diabetic nephropathy, pregnancy or lactation

### Retreatment criteria

The decision to repeat an injection after 4 weeks was based on the following criteria, which were not summative and each individual criteria when met, required re-treatment:

- BCVA worsening (by LogMar 0.10) from previously recorded maximal response post intravitreal injection
- Increase in CMT by 100 microns
- Appearance of Intra-retinal fluid

### Decision for re-treatment

All patients were strictly followed up at four weekly intervals and they underwent screening for re-treatment criteria using LogMAR chart BCVA measurement and SD OCT using a “tracking on” mode for comparability of measurements at each visit. The retinal OCT biomarkers were read by two experienced retinologists. The re-treatment decision was taken by another ophthalmologist who was blinded to the patient identification data and was only presented the re-treatment criteria measurements. These measures reduced bias to a great extent.

### Examination & data collection methods

All patients underwent a detailed ophthalmic evaluation on each visit with measurement of BCVA using i-Chart HD Smart (Appasamy Associates, Chennai, India), assessment of contrast using Pelli Robson Chart (Heidelberg inc, Heidelberg, Germany) and a complete ophthalmic examination including slit-lamp examination, intraocular pressure measurement. Fundus fluorescein angiography (at first visit), OCT of macula using centre point fixation and autotracking (Spectralis, Heidelberg inc, Heidelberg, Germany). The OCT was analysed for correct segmentation, SRF, IRF & NSD by two experienced vitreoretinal surgeons.

### Detailed physical and systemic examination was done

Each patient was examined at 4 weekly interval from week 0 to week 52. Intravitreal injection of Brolucizumab was administered at 0 week, at a dose of 6.0 mg in study Group 1 and 3.6 mg in study Group 2. After that patients who met retreatment criteria at each visit were re-injected with Brolucizumab 6.0 mg in Group 1 & 3.6 mg in Group 2.

Study variables –socio-demographic variable like age & sex were taken.

Each eye received 6.0 mg or 3.6 mg Intravitreal injection of Brolucizumab under topical anesthesia. The injection was administered with 30-gauge needle that was inserted 4 mm posterior to the corneal limbus in phakic patients and 3.5 mm posterior to the corneal limbus in pseudophakic patients under sterile conditions. All patients received topical antibiotic ophthalmic solution 0.5% Moxifloxacin for 03 days. The fractionation was done using a single puncture-multiple syringe technique. The 0.23 ml vial can easily give a 0.05 ml dose (6.0 mg) and a 0.03 ml dose (3.6 mg).

### Intraocular inflammation monitoring

All eyes injected with Brolucizumab were observed on Day 1, Day 7 and Day 28 for occurrence of any ocular inflammation using the following grading system (From KITE & KESTREL):

- Grade 0: No adverse events
- Grade 1: Subconjunctival haemorrhage and pain not requiring oral analgesic
- Grade 2: Anterior chamber [AC] cells and flare less than or equal to two with no circumciliary congestion, or ocular pain requiring oral analgesic
- Grade 3: AC cells and flare more than two, circumciliary congestion, vitritis grade 1, and patient not requiring oral steroids for management
- Grade 4: Hypopyon in AC, synechiae formation, vitritis more than grade 2, optic disc oedema/hyperaemia, retinal vascular sheathing/haemorrhages in retina in addition to pre-existing CNV

Once an IOI occurred the patients were monitored using Optos UWF Color picture, autofluorescence and FFA when indicated.

### Statistical analysis

All the data variables were compiled and entered in a Microsoft excel (Microsoft Excel 2021) sheet. The data was analysed with SPSS version 22 (IBM Corp., Armonk, NY, USA). The Percentage, mean and standard deviation were calculated for demographic and base line data. For qualitative data Chi-square test was used to compare the variables. For quantitative data independent t-test was used for comparing the mean of different groups and paired t-test was used to compare mean value of same groups. For related samples Friedmann’s two-way analysis of variance by rank was also done. The level of significance was fixed at < 0.05. To define the lower and upper boundaries 95% confidence interval was used. Bar diagrams were used to present the data.

### Informed consent procedures

All the investigations & procedures done in this study were safe for patients. An Informed consent proforma was prepared and informed consent was taken from every patient in English & spoken language of the patient, participating in the study. The subjects were informed about this research project sufficiently to their satisfaction & patient information sheet was provided to them regarding the drug profile. The subjects were free to opt out of the study at any point of time.

We declare that all participants gave consent to participate as well as consent to publish the data generated.

### Ethics & CTRI clearance

Institutional scientific and institutional ethical committee approval was taken for the study. Study was registered with clinical trials registry –India (CTRI ref no. CTRI/2023/06/054105)

It is declared that all guidelines as per declaration of Helsinki and good clinical practice guidelines were followed.

## Results

This was a prospective, randomized, double blind study conducted at the retina clinic in the department of ophthalmology at an apex institute of India.

### Demographic profile

The mean age of study participants in the 6 mg group was 63.7** ± **6.3 years and 3.6 mg group was 63.2** ± **6.3 years.

The gender distribution between the two groups was equally matched with 26 males and 15 females in both groups.

**Fig. 1:**
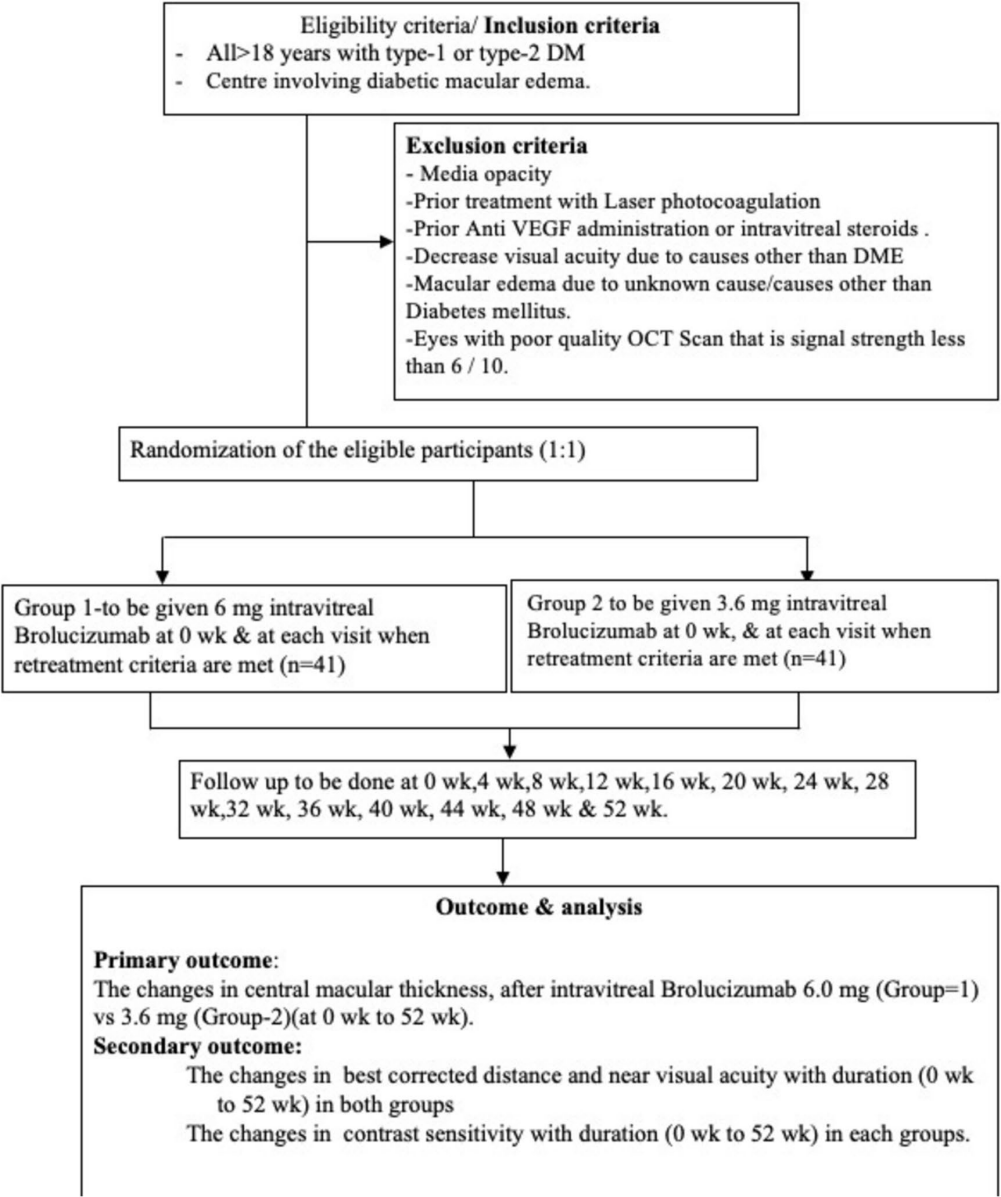
The consort/study flow diagram of the study

Laterality: In our study 24 eyes in 6.0 mg group were right eyes and 17 were left eyes and a similar distribution was present in 3.6 mg group also Fig. 1.

### Comparison of best corrected visual acuity (BCVA)

**Fig. 2:**
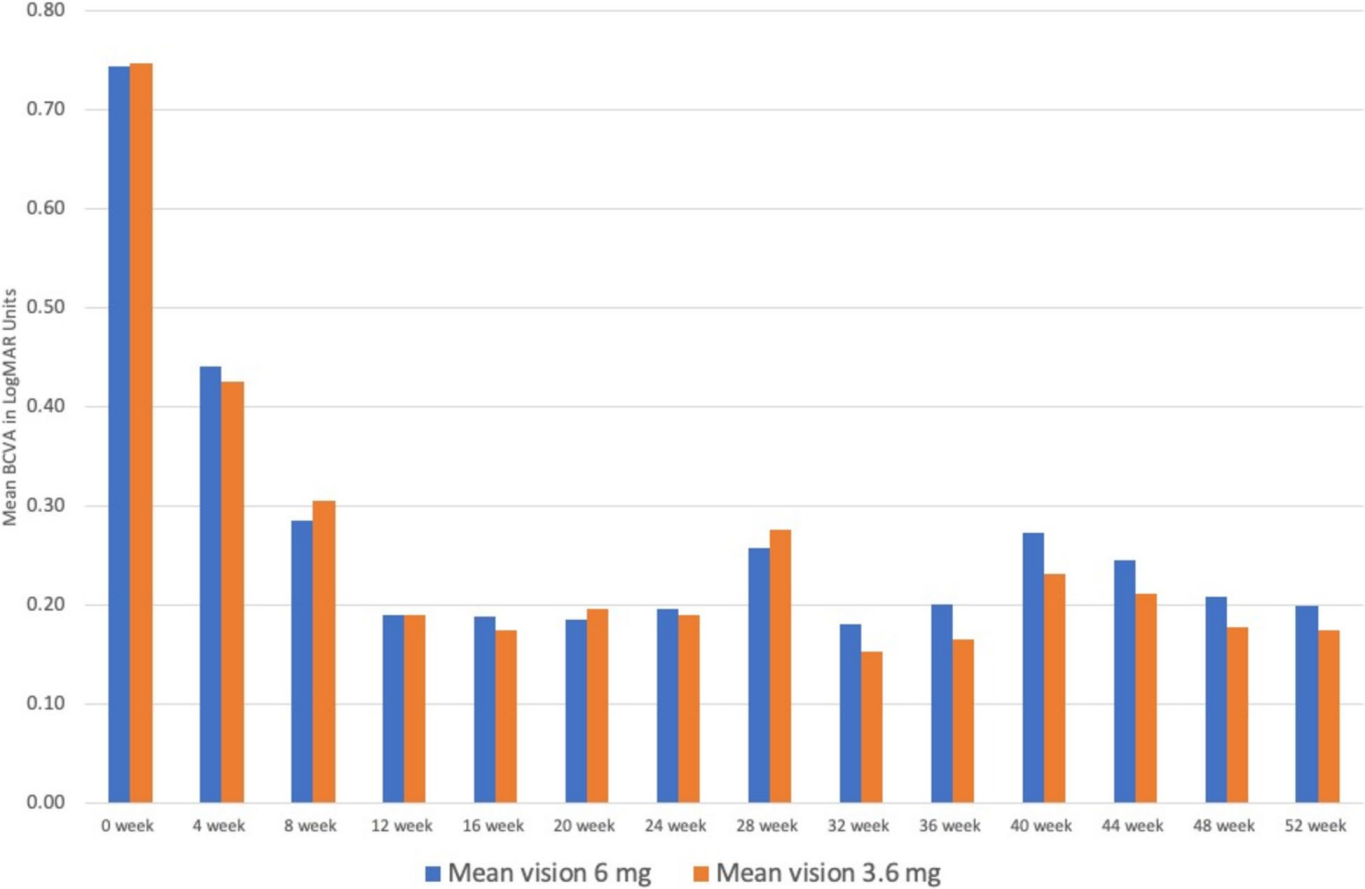
Mean BCVA amongst the two groups at each week

The changes in the visual acuity from 0 to 52 week in both the groups has shown mean change of 0.647 in group receiving 6.0 mg Brolucizumab and 0.698 in group receiving 3.6 mg of Brolucizumab injection respectively with p-value of 0.58, which shows that there is no statistically significant change in the visual acuity between both the groups as shown in Fig. 2.

### Comparison of central macular thickness (CMT)

**Fig. 3:**
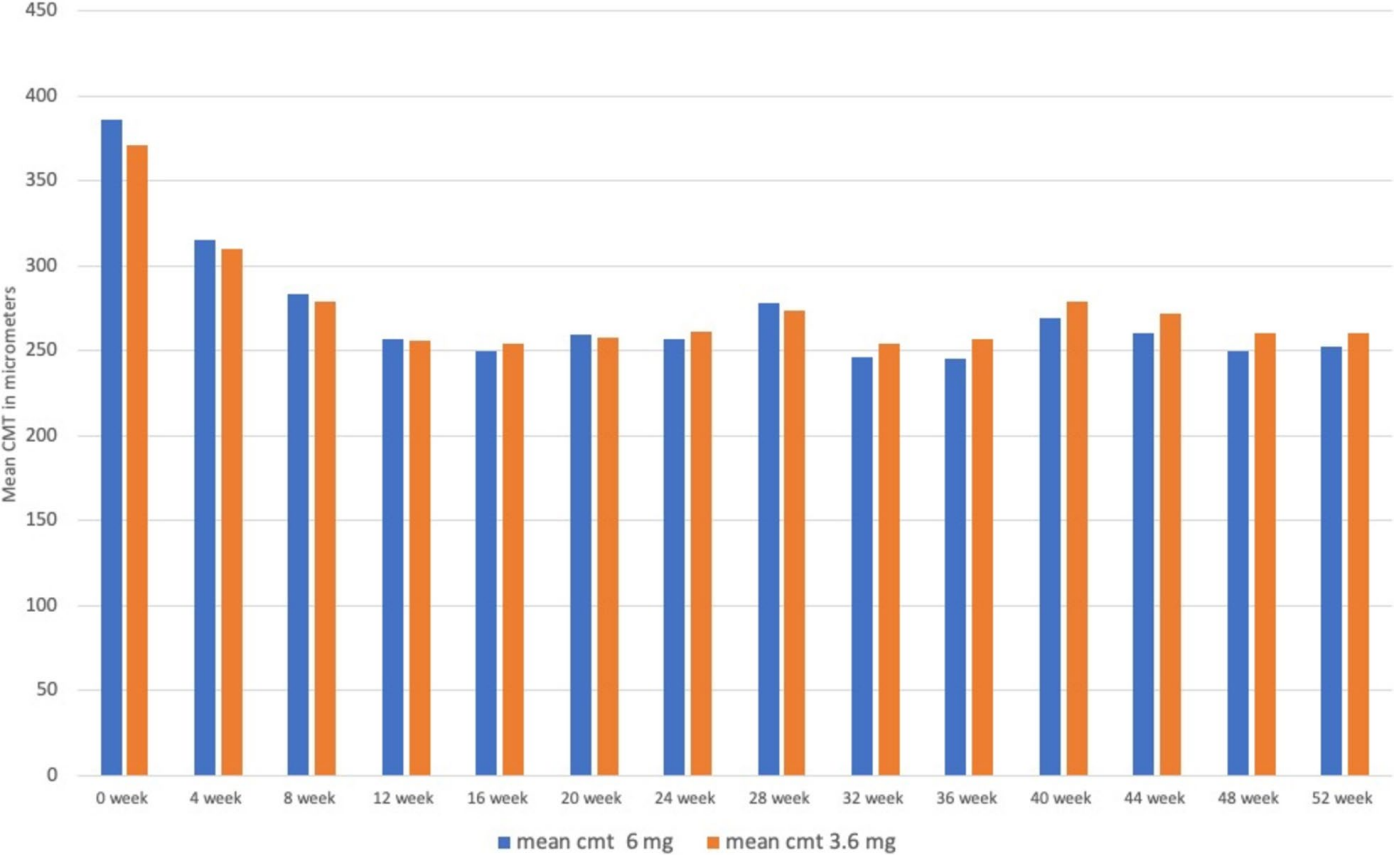
Meant CMT on OCT between both the groups at each week

The changes in the CMT from 0 to 52 week in both the groups has shown mean change of 133.2 µm (μ) in group receiving 6.0 mg Brolucizumab and 110.6 μ in group receiving 3.6 mg of Brolucizumab injection respectively with p-value of 0.58. Which shows that there is no statistically significant change in the change in macular thickness between both the groups as shown in Table 1 & Fig. 3.

**Table 1:** The mean CMT change in both the groups from week 0 to week 52

Week	Mean CMT 6.0 mg	Std dev CMT 6.0 mg	Mean CMT 3.6 mg	Std dev CMT 3.6 mg
0 week	386.04878	64.5561582	371.463415	47.1747271
4 week	315.512195	56.1961395	310.146341	54.4828234
8 week	283.341463	50.3873048	279.243902	51.6264373
12 week	257.439024	38.0519702	255.780488	37.0759438
16 week	250.365854	42.0504198	254.707317	44.7483206
20 week	260.121951	55.5036914	257.585366	54.3290786
24 week	257.268293	56.4065707	261.268293	57.368556
28 week	278.146341	53.0450568	273.97561	51.1060113
32 week	246.073171	35.1001355	254.682927	46.3699466
36 week	245.878049	36.2044163	257.317073	50.0796561
40 week	269.170732	51.514999	279.170732	62.3758376
44 week	260.560976	42.9651305	272.195122	59.9167003
48 week	250.170732	39.5081653	260.780488	53.2937671
52 week	252.804878	42.8066698	260.853659	48.8306057

“p” value at 0 weeks was 0.82924 and at 52 weeks was 0.84529

**Fig. 4:**
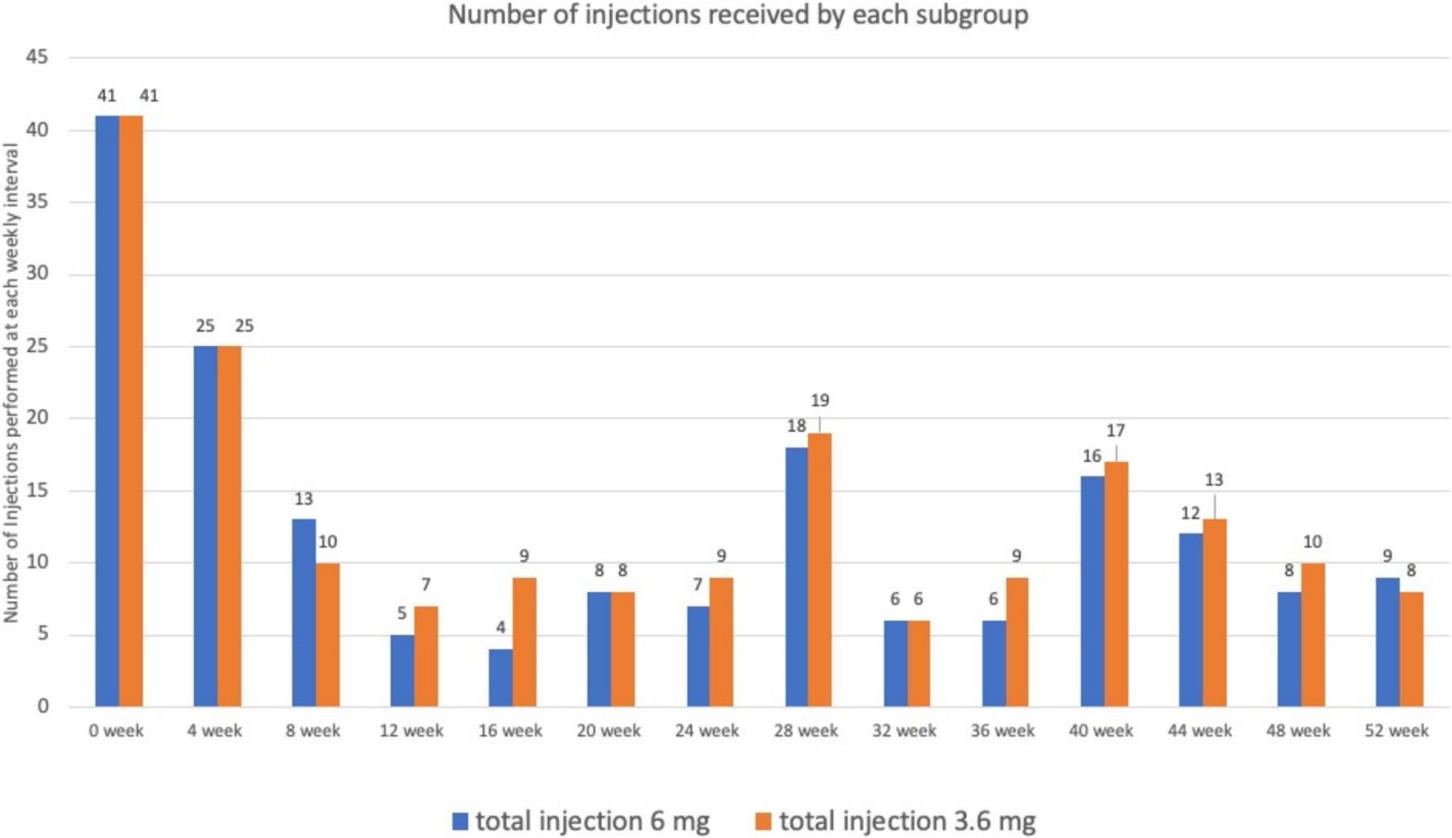
Bar diagram of number of injections received by each subgroup at 4 weekly intervals

Comparison of number of injections per participant in each group has been shown in Fig. 4.

### Re-injection interval between the two sub groups

As all the participants were given first injection at zero week, the number of reinjection events during the next 52 weeks were calculated and divided by 48 weeks to obtain the inter-injection interval. The interval was 15.56 weeks for 3.6 mg subgroup and 14.21 weeks.

Table 2 shows omparison of diabetic macular edema on Fundus Fluorescien Angiography at initiation between both groups.

**Table 2:** FFA characteristics of DME in each gp at baseline

FFA-showing focal/diffuse leakage at fovea	6.0 mg		3.6 mg		p value
	Count	%	Count	%	
No	3	7.3%	3	7.5%	0.975
Yes	38	92.7%	37	92.5%	
Total	41	100.0%	41	100.0%	

### Comparison of contrast

**Fig. 5:**
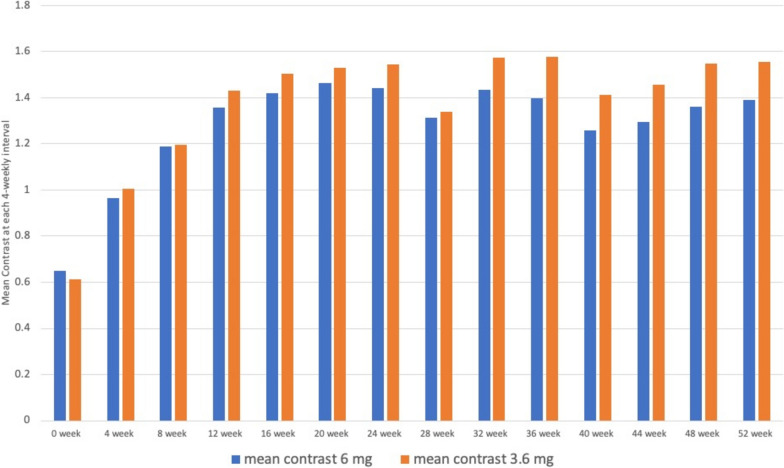
Graphical depiction of the mean contrast values between the two subgroups at each weekly interval

In the present study, no statistically significant difference was found in contrast at different time intervals as shown in Table 3 & Fig. 5.

**Table 3:** Comparison of Contrast at different time interval between both groups

Week	Mean contrast 6.0 mg	Std dev contrast 6.0 mg	Mean contrast 3.6 mg	Std dev contrast 3.6 mg
0 week	0.65121951	0.30403285	0.61341463	0.28107481
4 week	0.96463415	0.4033057	1.00609756	0.45637089
8 week	1.1902439	0.36438639	1.19756098	0.45372778
12 week	1.35609756	0.34860349	1.42926829	0.40911117
16 week	1.41829268	0.36379185	1.50365854	0.42328924
20 week	1.46463415	0.37454037	1.52804878	0.44708064
24 week	1.44268293	0.42581701	1.54512195	0.44311467
28 week	1.3125	0.47754044	1.33780488	0.57649593
32 week	1.43414634	0.35609673	1.57439024	0.33840101
36 week	1.39878049	0.34972986	1.57926829	0.35213201
40 week	1.2597561	0.43863702	1.41097561	0.50068855
44 week	1.29634146	0.41508888	1.45487805	0.41859958
48 week	1.3597561	0.41264687	1.54878049	0.39772601
52 week	1.38878049	0.37374587	1.55609756	0.43072542

“p” value at 0 weeks was 0.31728 and at 52 weeks was 0.28795

### Average vision improvement

**Fig. 6:**
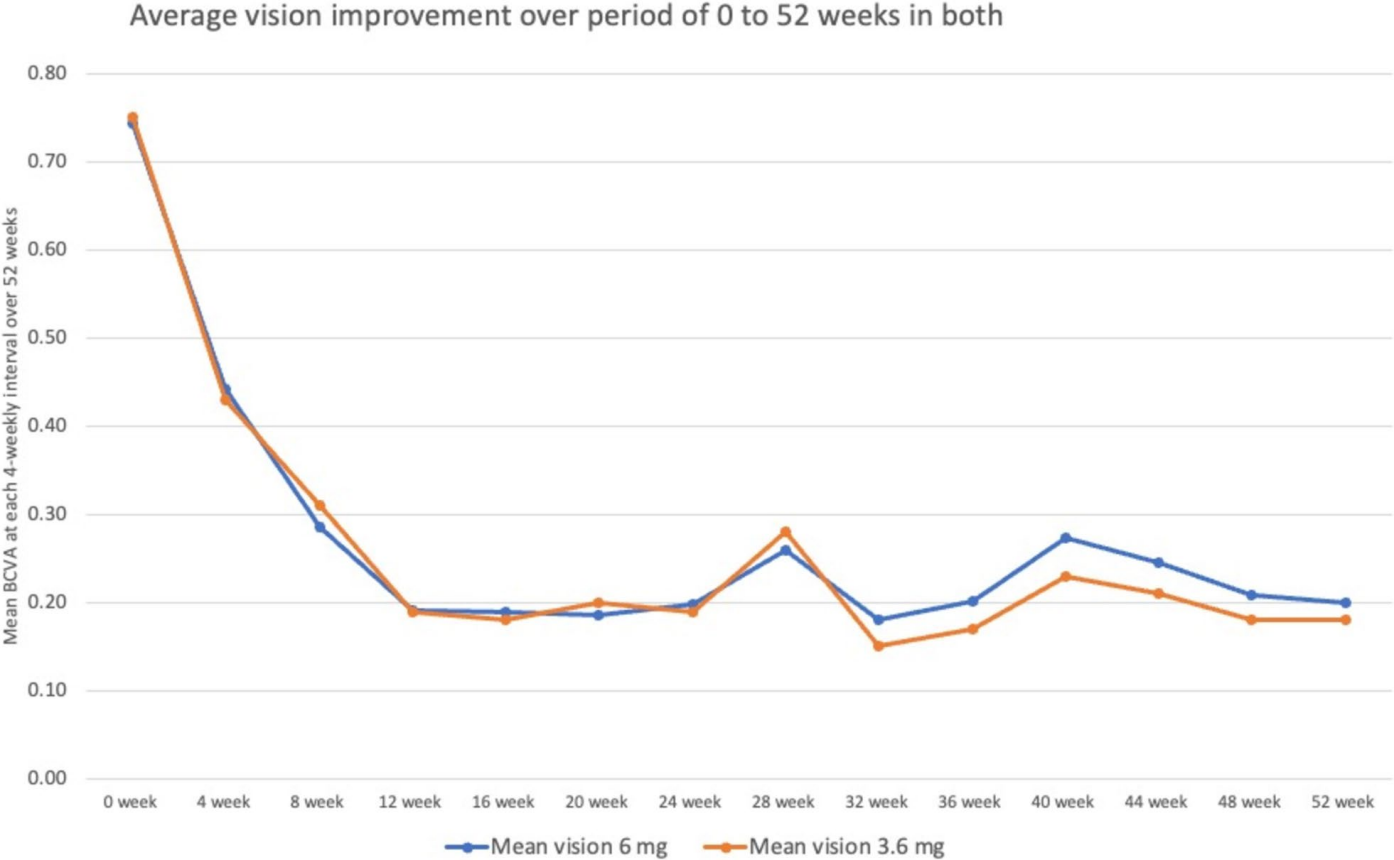
Average vision improvement over period of 0 to 52 weeks in both gp

The average vision at 4 weekly interval is shown in Fig. 6.

### Comparison of adverse events

**Fig. 7:**
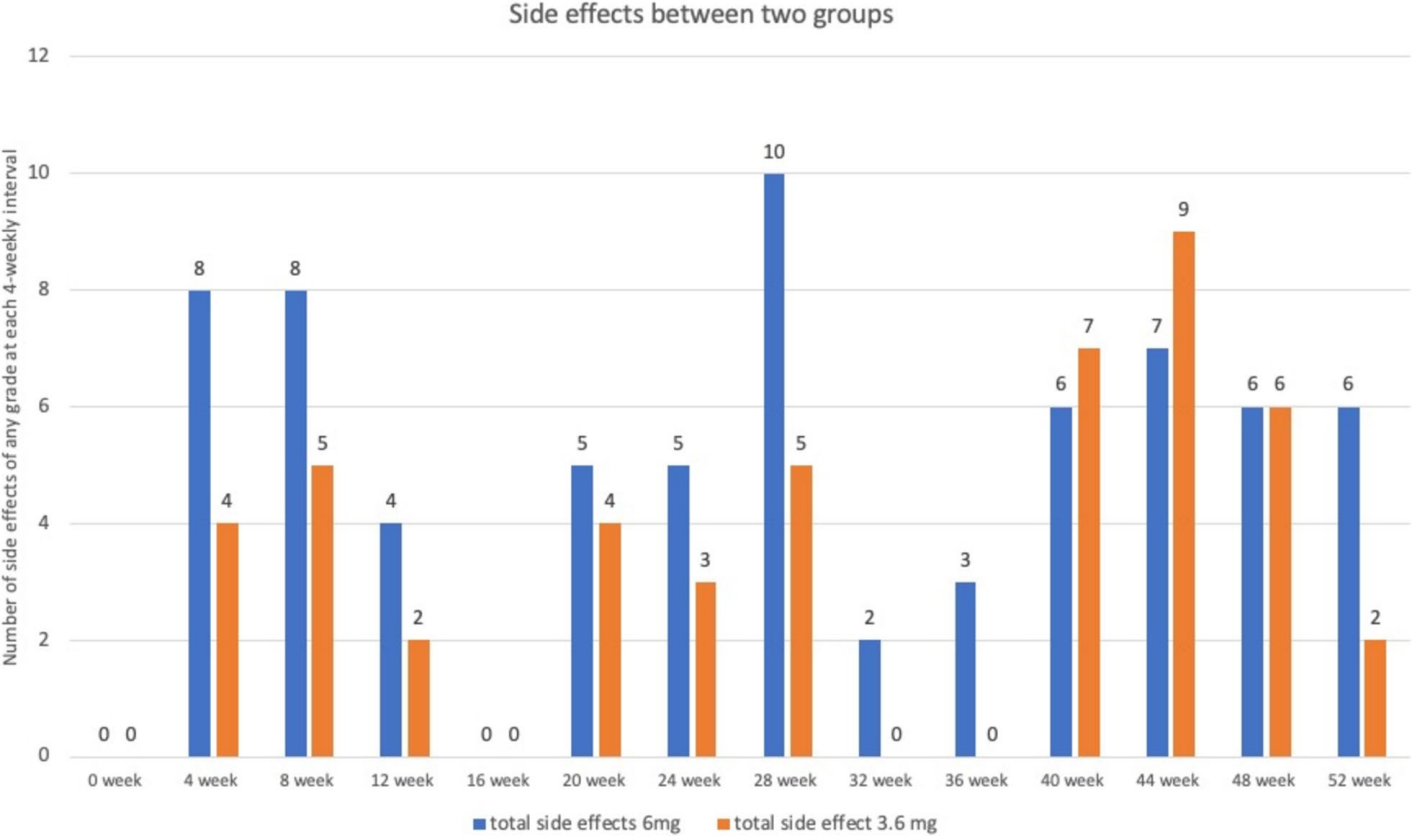
The total number of adverse events occurring per injection at 4 weekly interval (includes all grade 1 to Grade 4 events)

Figure 7 demonstrates all the side effects (Grade 1 to Grade 4) at each 4 weekly visit in each group. There was one grade 4 adverse events in the 6.0 mg group and one grade 4 adverse event in the 3.6 mg group. Both were managed with administration of topical steroids and posterior sub-tenon steroids. None of the patients ended with final BCVA at 52 weeks less than that at baseline.

Table 4 shows correlation between various study variables:

**Table 4:** Correlation of study variables and the conclusions drawn

S No	Pearson corelation coefficient	Variables	Conclusion
1	0.99593036	Intraretinal cystic space appearance and administration of injection	Very strong positive correlation; almost perfect linear relationship
2	0.374265823	Neurosensory detachment appearance and administration of injection	Moderate positive correlation; significant linear relationship
3	0.299495663	Appearance of traction on OCT and administration of injection	Moderate positive correlation; some linear relationship
4	0.18688659	Occurrence of side effects i and administration of injection	Weak positive correlation; slight linear relationship
5	0.770028998	BCVA LogMAR value and administration of injection	Strong positive correlation; strong linear relationship
6	0.803219329	Central macular thickness on OCT and administration of injection	Strong positive correlation; strong linear relationship
7	− 0.770993337	Absolute Contrast value and administration of injection	Strong negative correlation; strong inverse linear relationship
8	0.917108963	BCVA LogMAR value and CMT on OCT	Very strong positive correlation; very strong linear relationship
9	− 0.897550295	BCVA LogMAR value and & Absolute Contrast value	Very strong negative correlation; very strong inverse linear relationship
10	− 0.832077573	Absolute Contrast value & CMT on OCT	Strong negative correlation; strong inverse linear relationship

A very strong correlation was seen between presence of intraretinal fluid (IRF) in the form of cystic spaces on OCT and administration of injections. On most occasions when a decision to reinject was taken IRF was present. Similarly, there was a strong correlation between administration of injections and drop in BCVA & increase in CMT on OCT and reduction in absolute contrast values.

The correlation between appearance of neurosensory detachment or presence of traction on OCT did not correlate strongly with the decision to reinject.

The appearance of side effects did not show strong correlation with the administration of injections.

All data generated or analysed during this study are included in this published article [and its supplementary information file number 1].

## Discussion

The primary reason for loss of central vision and contrast in diabetic retinopathy is diabetic macular edema [9]. The emergence of anti-VEGF drugs paved a treatment protocol of managing DME using repeated injections of anti-VEGF [10]. This requires constant monitoring of the patient using OCT at 4 to 6 weeks intervals and repeated injections of anti-VEGF which lead to an increasing economic burden both in terms of the cost of drug, the hospital costs in administering the drug and the loss of manpower during the treatment [11]. Efforts have been made to discover longer acting anti VEGFs. The drug Aflibercept was introduced about two decades ago with the promise of 08 weeks duration of action, however, the real world experience shows durability between 6 to 7 weeks [12]. The drug Brolucizumab was introduced about 6 years ago and adequate research was available regarding the efficacy and durability of Brolucizumab. However, a certain question regarding using a lower dose of Brolucizumab in treating diabetic macular edema remained unanswered. The genesis of the idea of using a lower dose of anti-VEGF in DME arises from The US FDA approval of a lower dose of Ranibizumab i.e. 0.3 mg in treating patients of DME [13]. The researchers who have done pioneering research on Brolucizumab in the KITE and KESTREL study have also used a lower dosage subgroup in the KESTREL study [14].

We wanted to examine the impact of a lower dosage of Brolucizumab i.e. 3.6 mg compared to 6.0 mg. The dose of 3.6 mg was chosen because the commercially available solution of Brolucizumab gives 6.0 mg in 0.05 ml and the graduations available for titration of drug were only available at 0.01 mill graduations. Hence, we decided to use 0.03 ml of this solution i.e. 3.6 mg as the lower dose subgroup in our study.

The 2 subgroups which were chosen were well matched for age and gender as well as laterality of the eyes. The baseline characteristics of vision, contrast, central macular thickness were also well matched between the two groups. We went ahead with using a pro re nata regimen for management of DME [15], using the pre-defined retreatment criteria which were similar to those used by the KITE & KESTREL study. An important consideration was the treatment naïve status of these patients for enrolment in the study to avoid the loss of first dose impact of anti-VEGF in DME.

The best corrected visual acuity improved significantly from baseline in both the subgroups, however when the change in best corrected visual acuity from baseline was compared in both the subgroups there was no statistically significant difference between the two. The change in BCVA was 0.54 LogMAR (27 letters) in 6.0 mg and 0.59 (29.5 letters in 3.6 mg group. This is better than the 100 week results of KITE (+ 8.8 letter) and KESTREL (+ 10.9 letters) [14].

Similarly, when the central macular thickness measurements were observed from baseline they improved significantly in both the subgroups, however the change in central macular thickness from baseline when compared between the two subgroups did not show statistically significant difference. This indicates that the efficacy of both dosages i.e. 3.6 mg as well as 6.0 mg is almost similar in cases of DME when treated on prn basis. Our results were almost similar to those of Kestrel study [16].

To analyse the safety of the 2 dosages of Brolucizumab, we proactively looked for any ocular adverse effects at each 4 weekly interval. The total number of grade 3 and grade 4 adverse events which occurred in the 2 subgroups were almost similar and did not show any statistically significant difference. We observed two grade 4 adverse events throughout the study, out of which one occurred in 3.6 mg subgroup and one occurred in 6.0 mg subgroup [17]. This indicates that the safety of both the dosages is almost similar as has been reported by KESTREL study [14].

The inter-injection interval obtained in the two subgroups over 52 weeks interval was even more promising than the manufacturer recommendation of 12 weekly dosing. This gives strength to the idea of 12 weekly follow up after injection of Brolucizumab in DME [18].

We also actively monitored the reasons for re-treatment being done in each arm. We found that almost in 98% trigger events the reason was a fall in BCVA of 0.1 from previously achieved BCVA after the injection [19]. This opens up the discussion for home based monitoring of the DME and identifying patients requiring re-treatment. This will tremendously reduce the burden of hospital visits for the patients as well as save precious health resources.

The results from this study thus show that the 3.6 mg dosage is similar in efficacy to 6.0 mg Brolucizumab in the treatment of Diabetic Macular edema with a similar safety profile. This has important consequences for the financial impact of this drug as it practically means that an ophthalmologist can safely deliver three dosages of 3.6 mg (0.03 ml) Anti-VEGF from a single commercially available vial of Brolucizumab 0f 27.6 mg in 0.23 ml. The adoption of such dosage would bring down the treatment costs by two thirds which will be a significant amount considering the overall cost of Anti-VEGF being administered in our country [11].

## Conclusion

The study has shown that both 6.0 mg and 3.6 mg dosages of Brolucizumab have similar efficacy and safety in management of treatment naïve DME, while providing durability of over 14–15 weeks in both subgroups. This will have significant impact on the costs involved in terms of reduction in the cost being incurred on procuring this Anti-VEGF and reduction in hospital visits by the patient.

## Limitation of the study

The study was conducted at a tertiary care hospital and may have suffered from patient selection bias. The study had a major limitation of predominant male participants compared to female participants.

## Supplementary Information

Supplementary Material 1.

## Data Availability

Datasets which were generated or analysed during the current study will be made available on request.
