# Supplementary figures and images for: Efficacy & safety of brolucizumab 6.0 mg versus 3.6 mg in diabetic macular edema
Source: Int J Retina Vitreous. 2025 Jan 13;11:6. doi: 10.1186/s40942-025-00628-x (PMC11727401; doi:10.1186/s40942-025-00628-x)

Appx A

MASTER CHART

6.0 MG GROUP


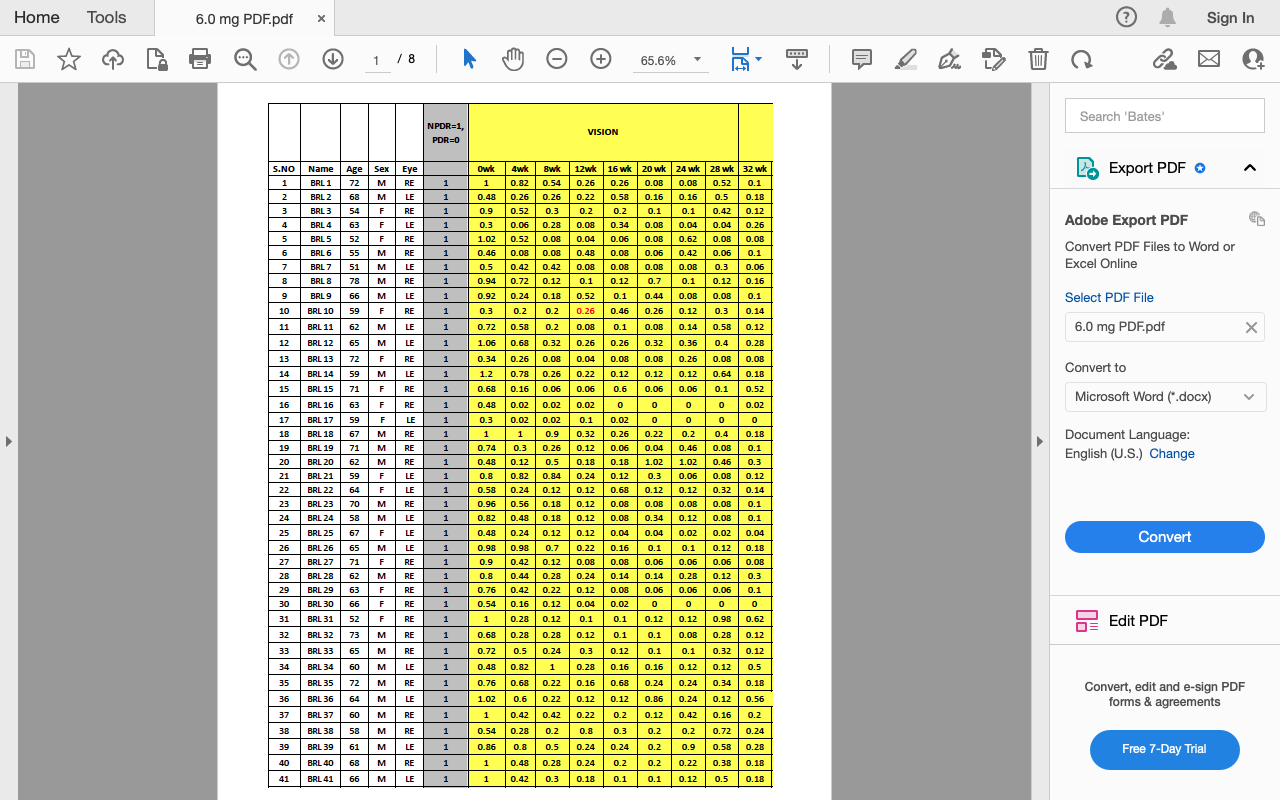


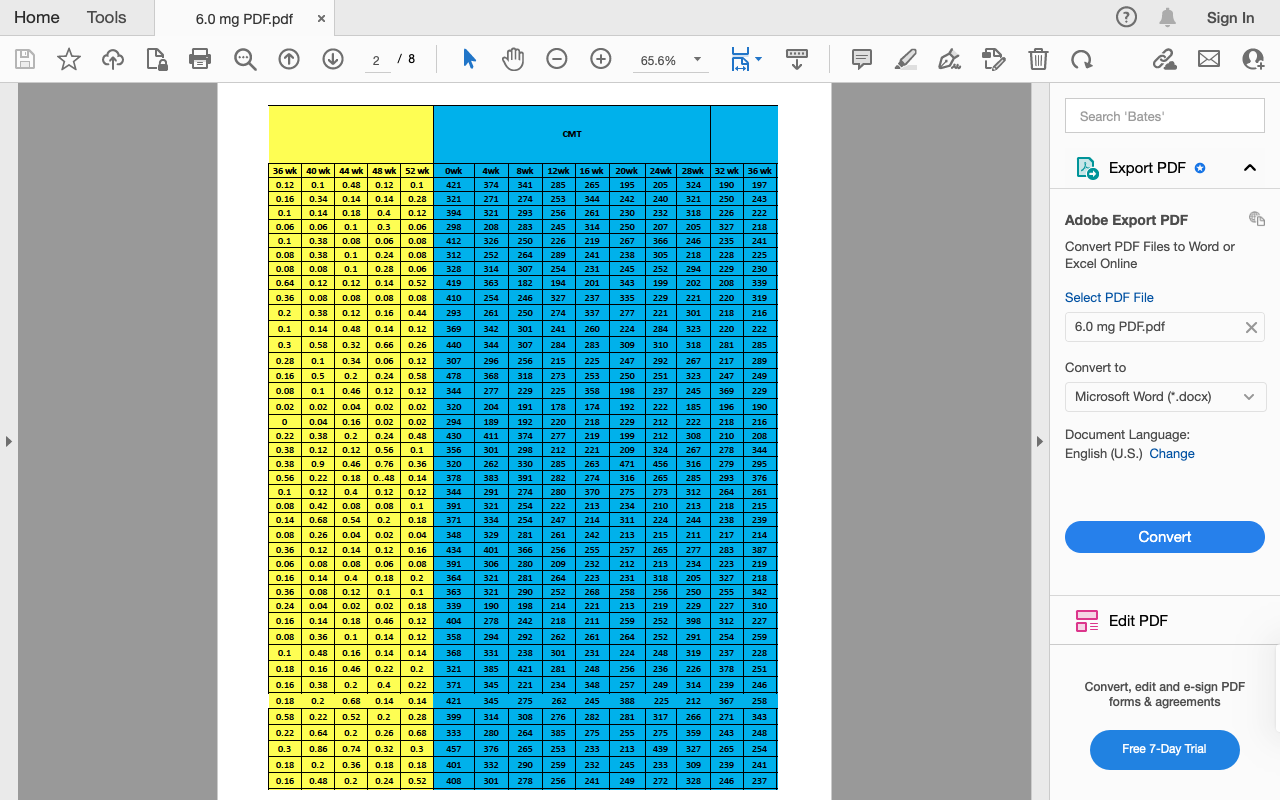


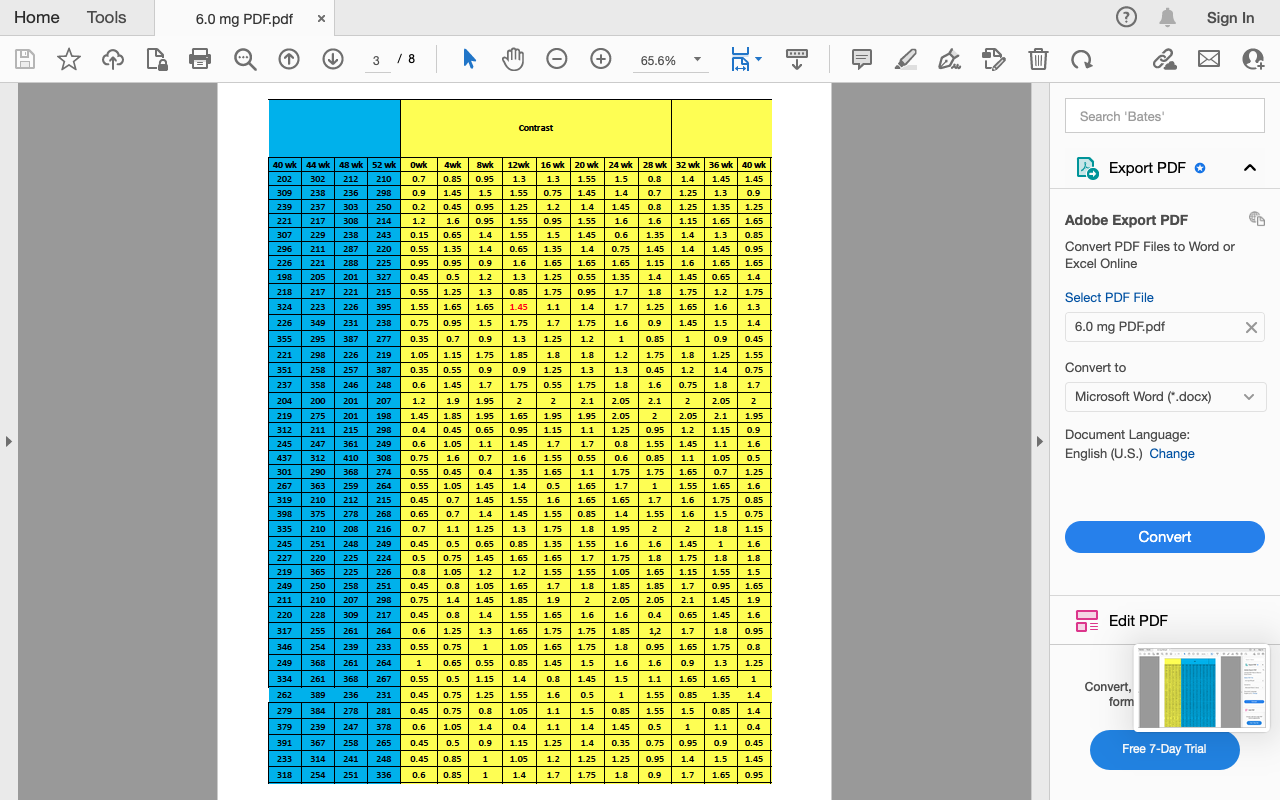


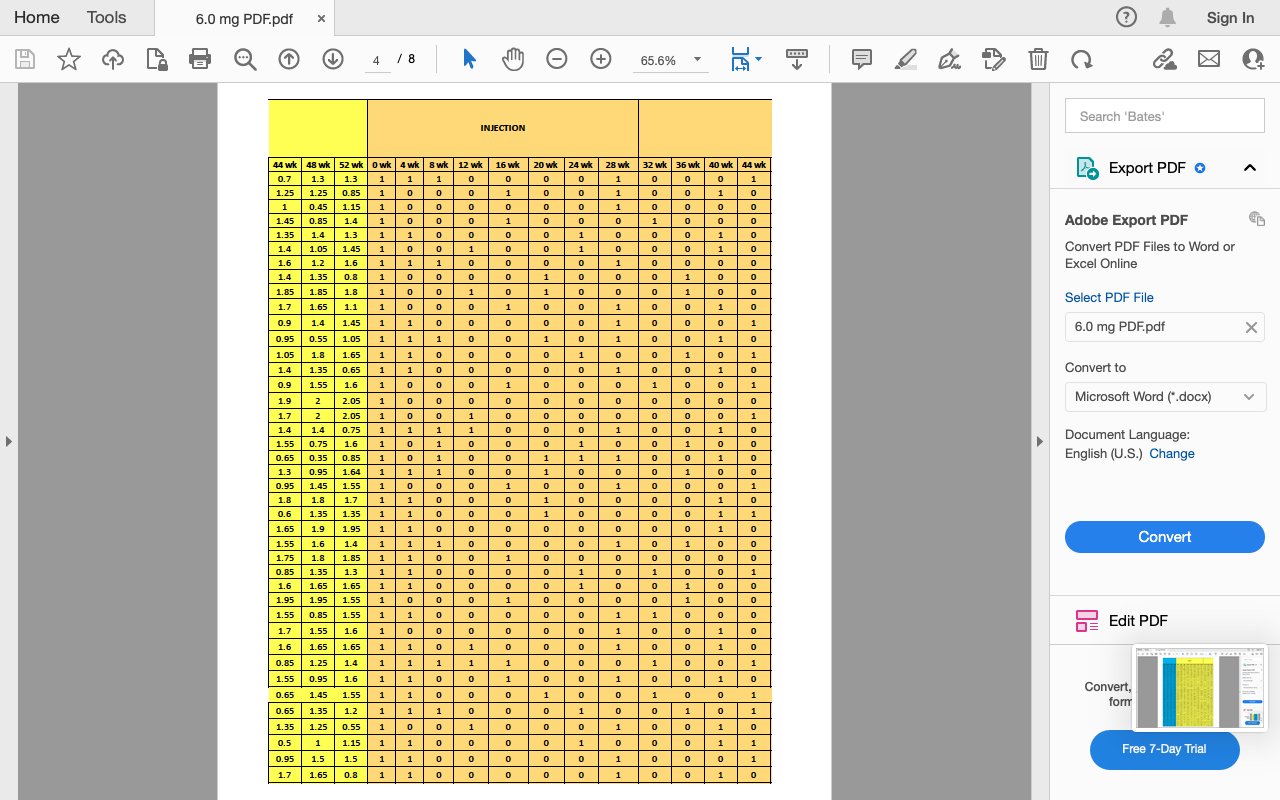


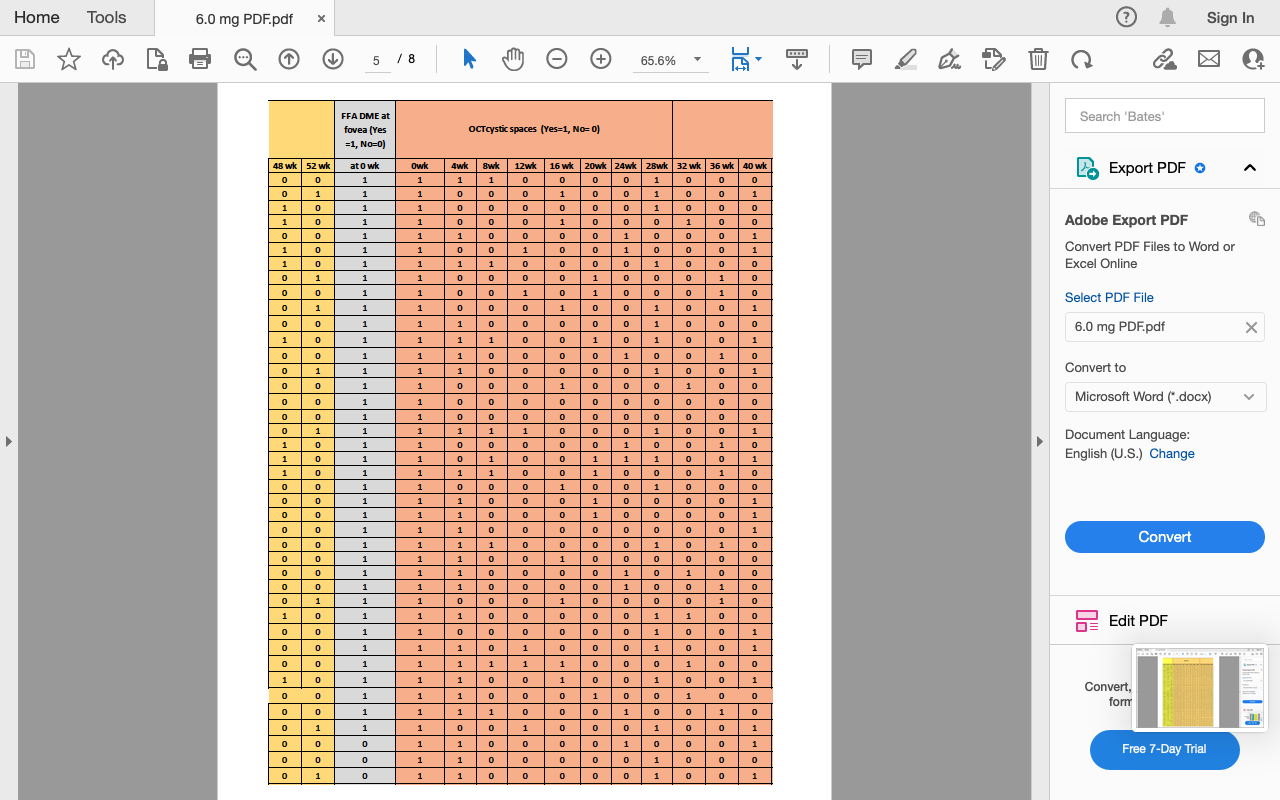


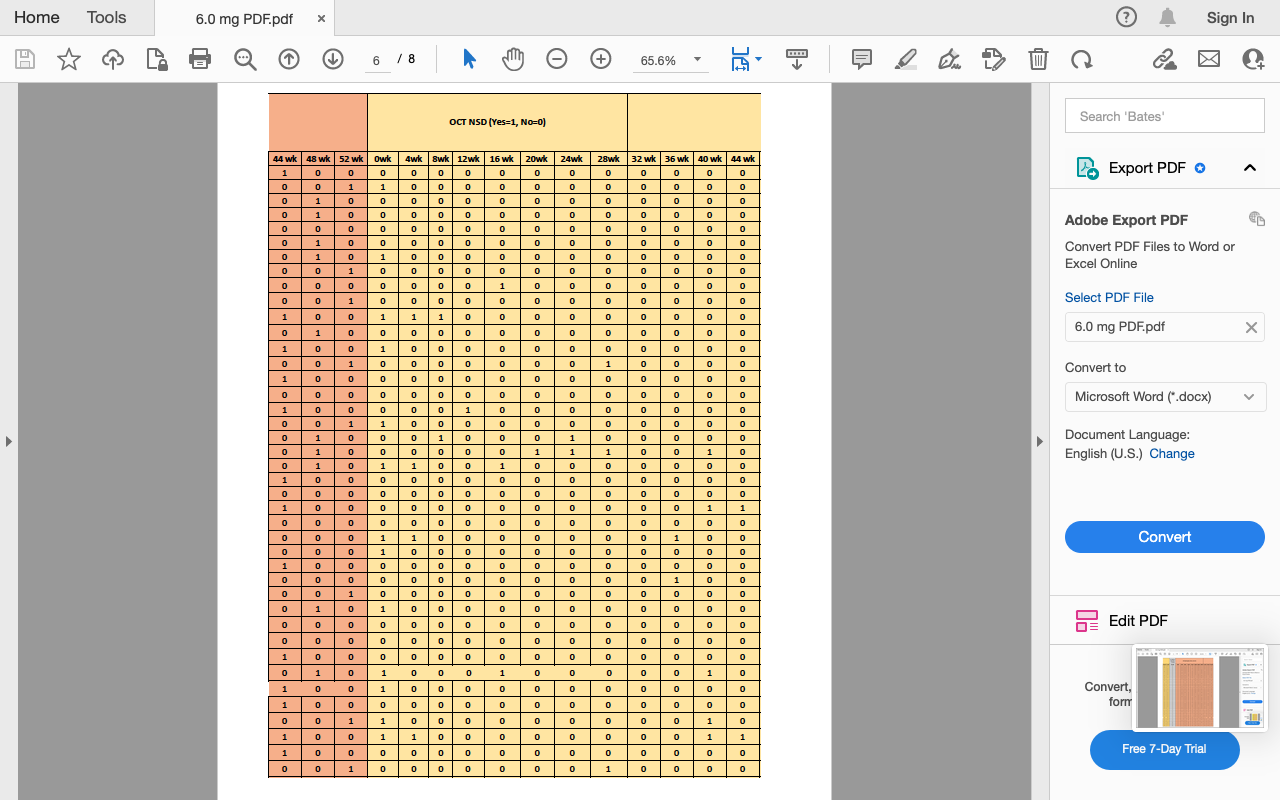


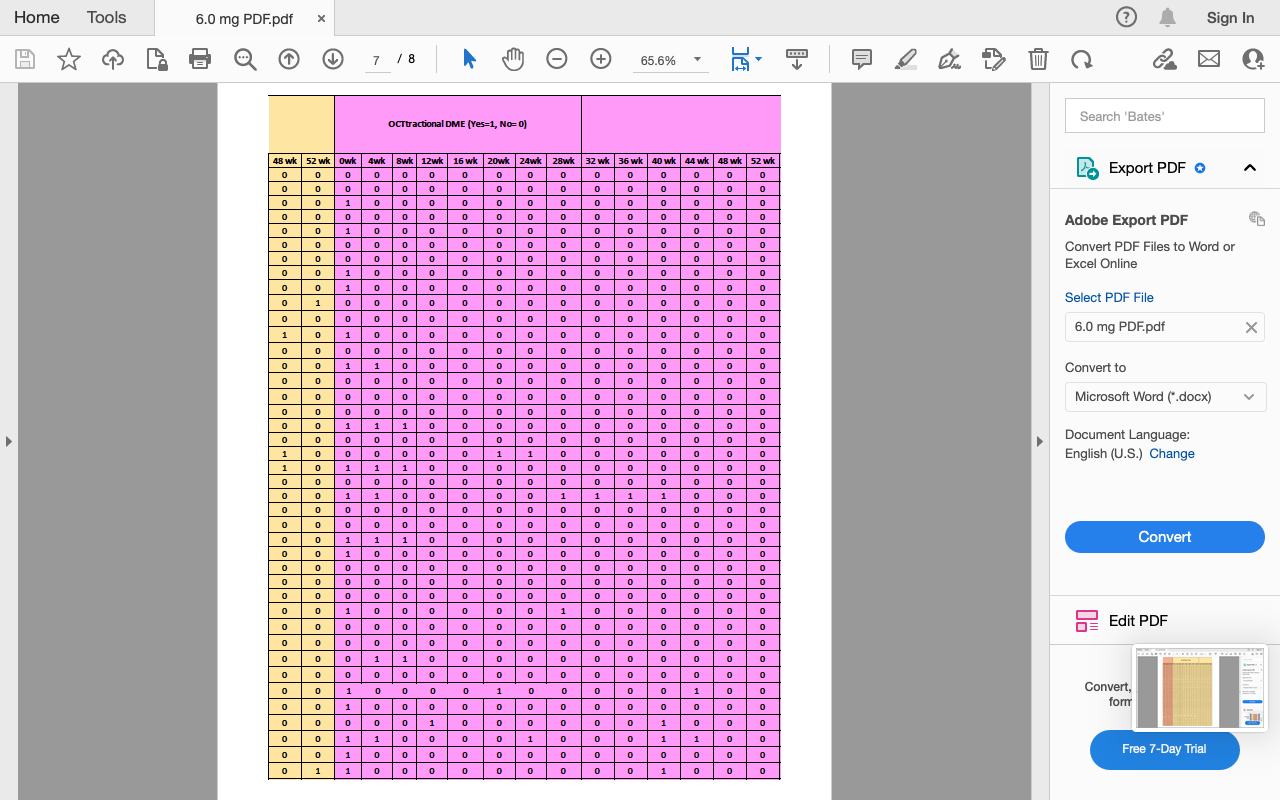


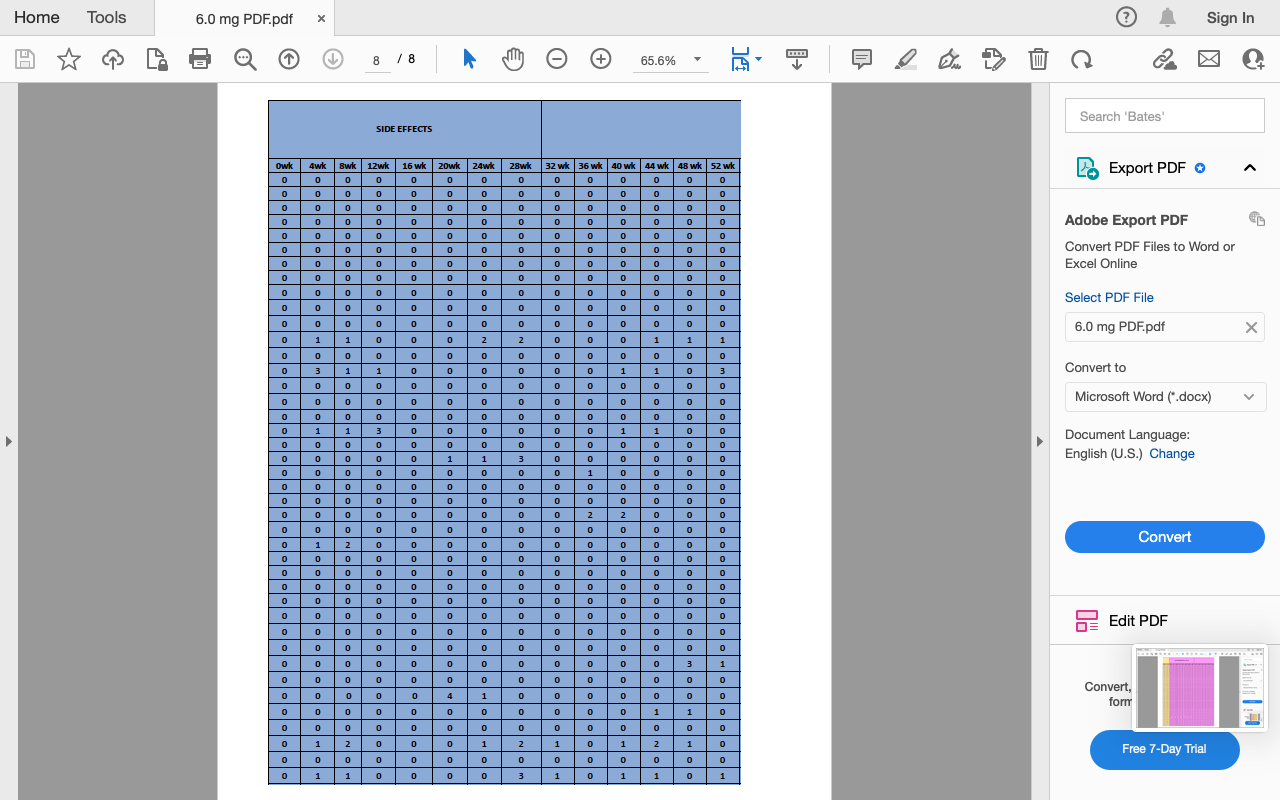


3.6 MG GROUP


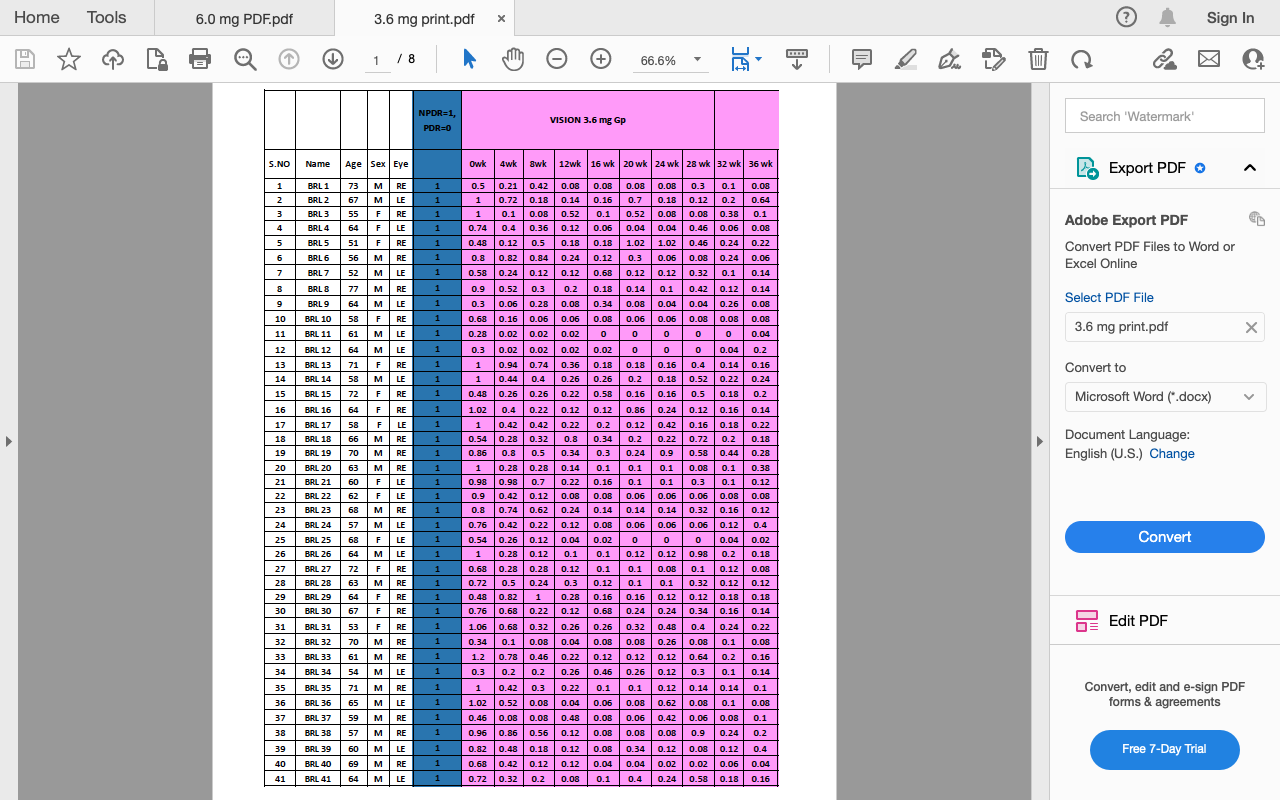


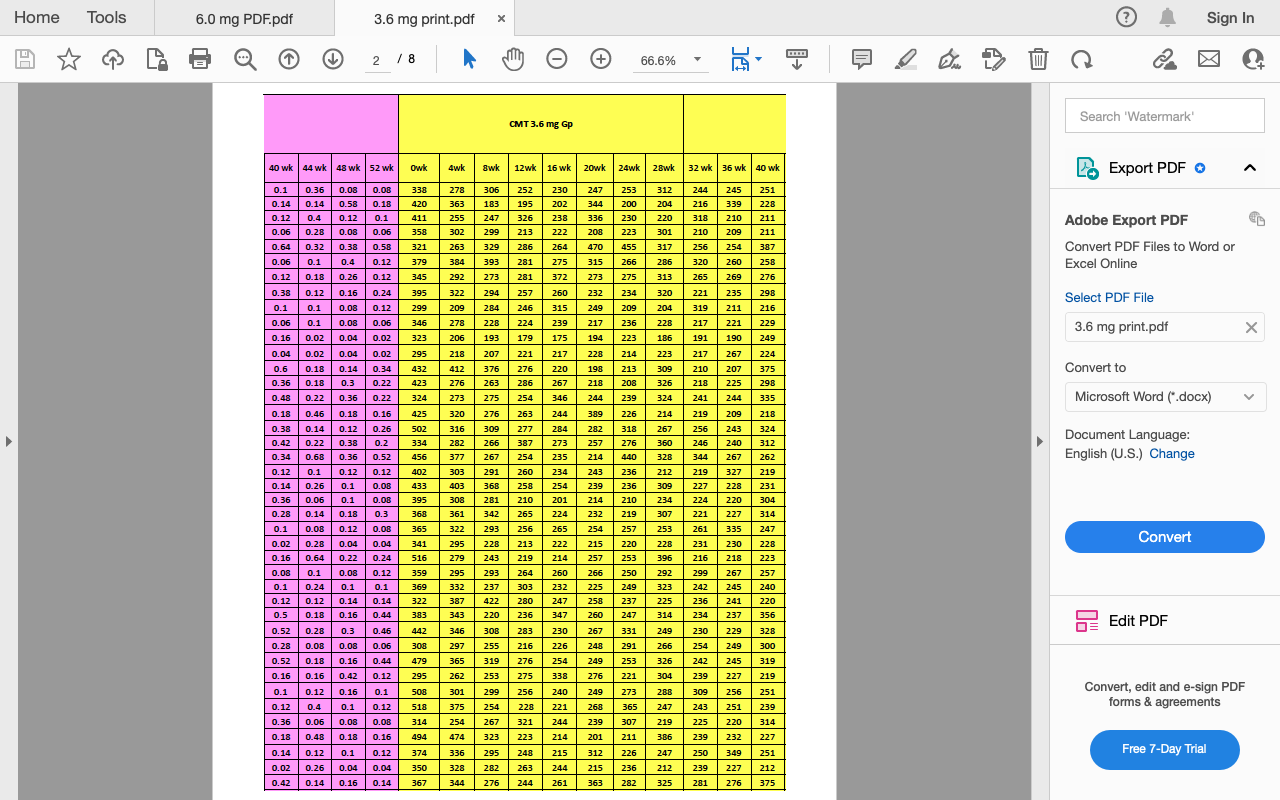


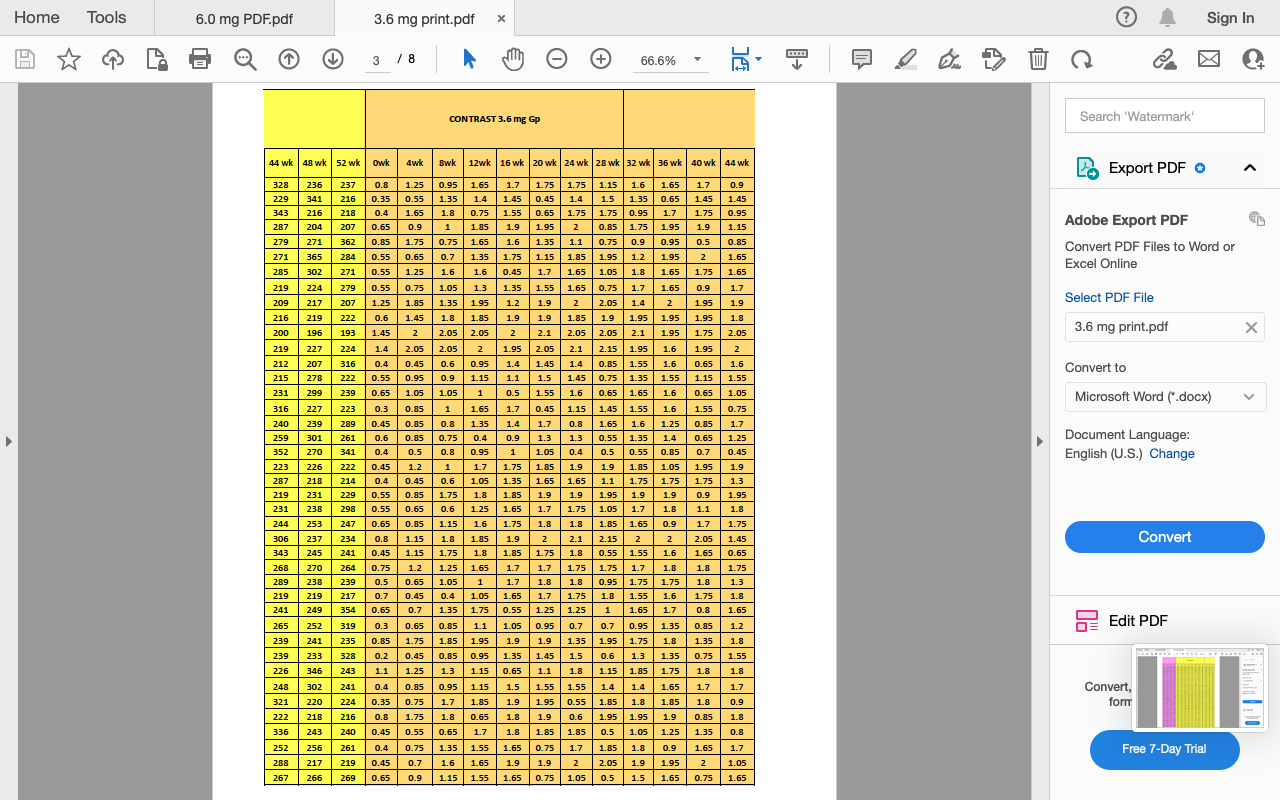


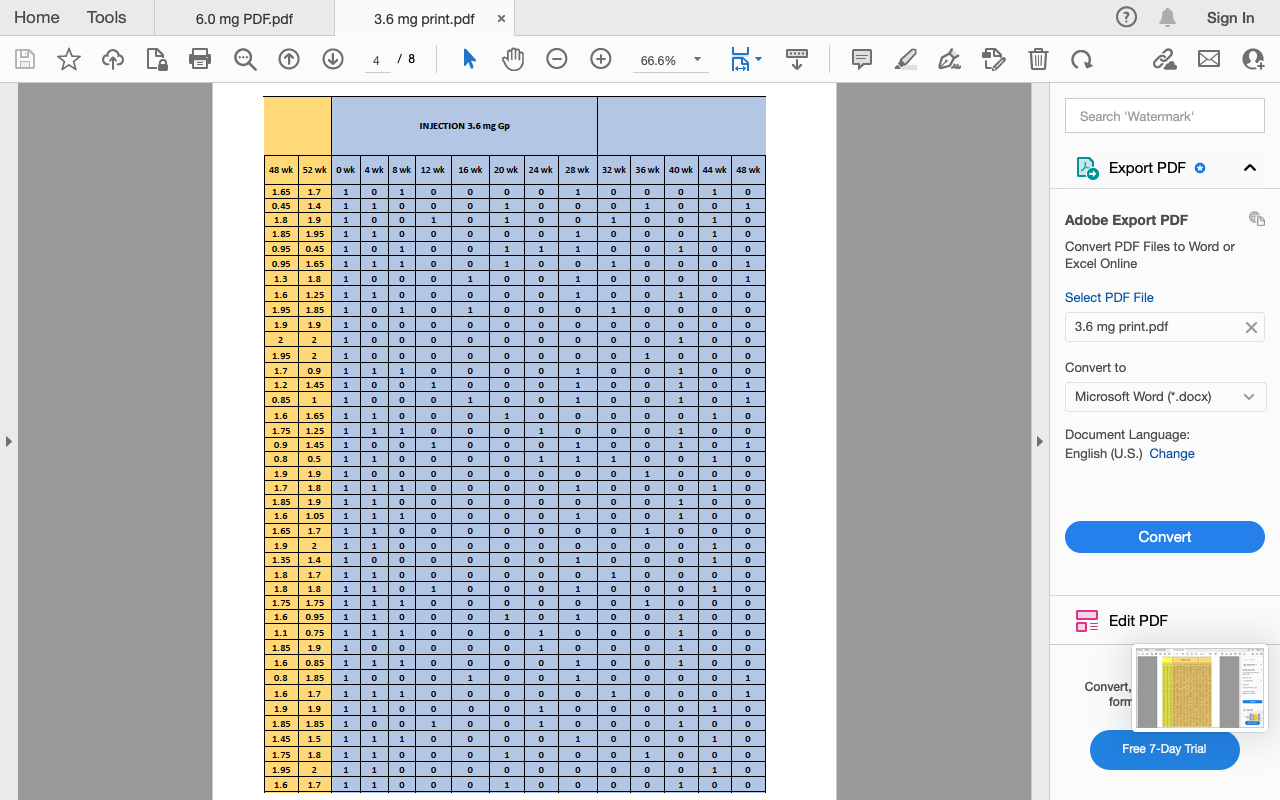


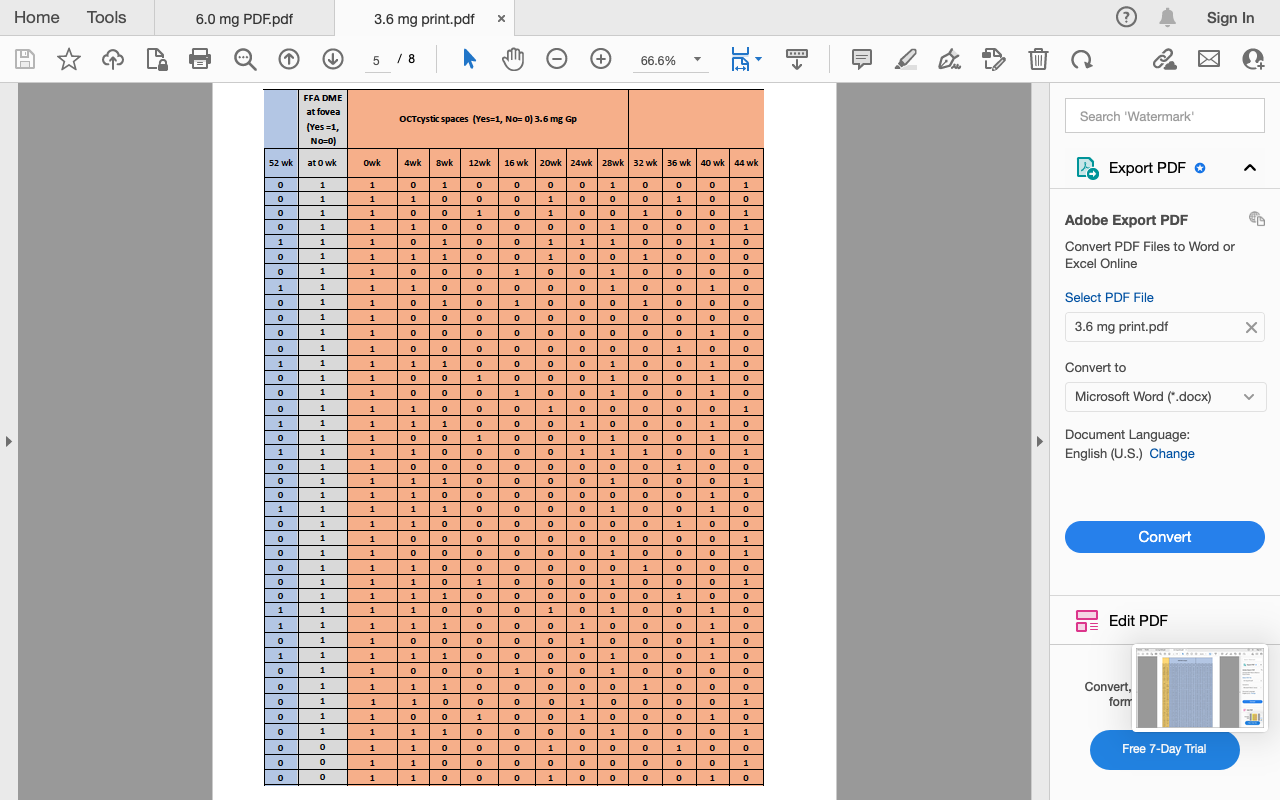


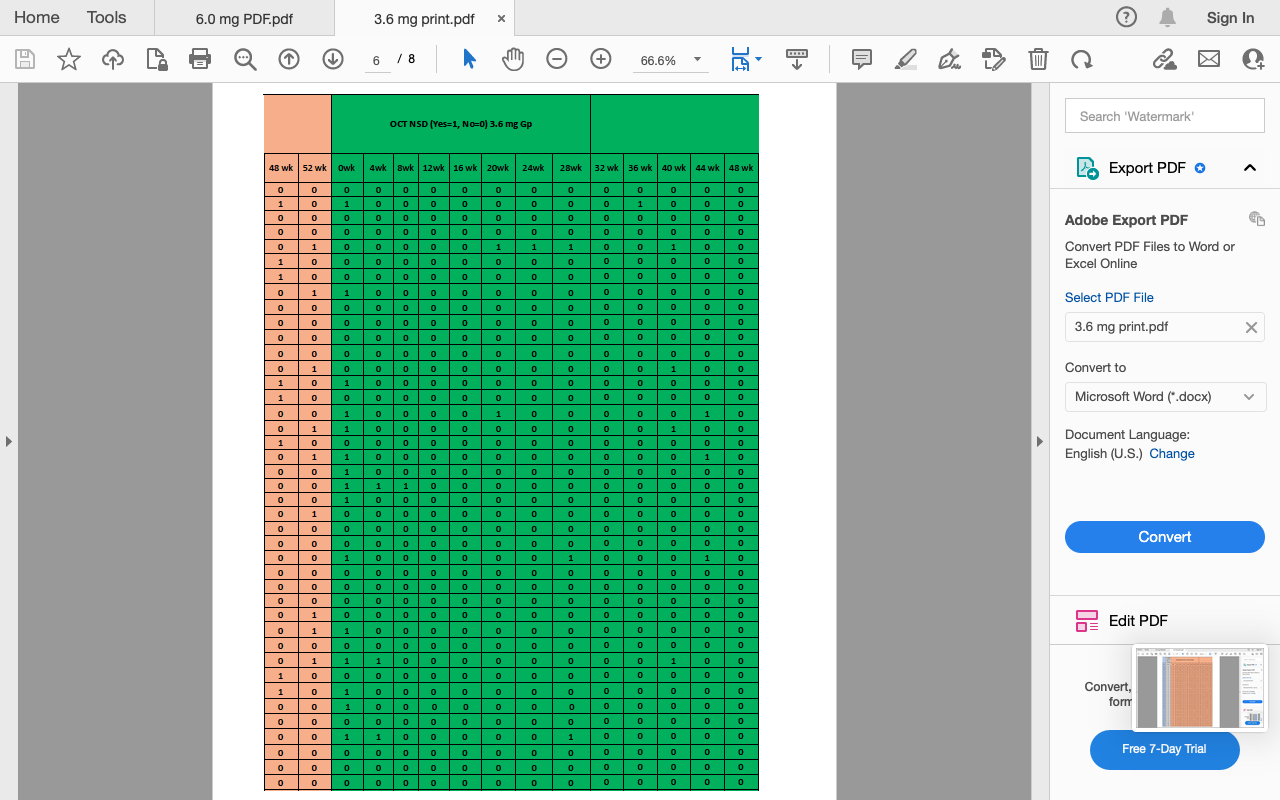


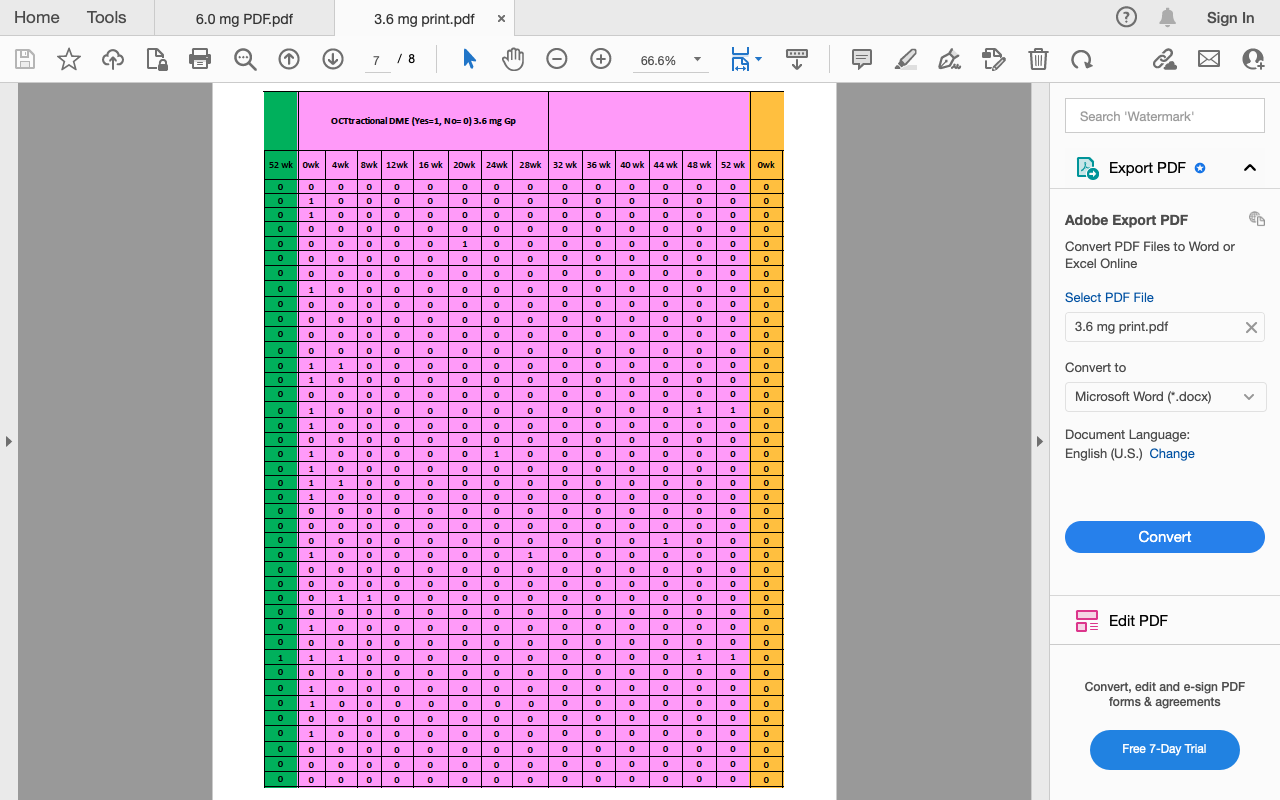


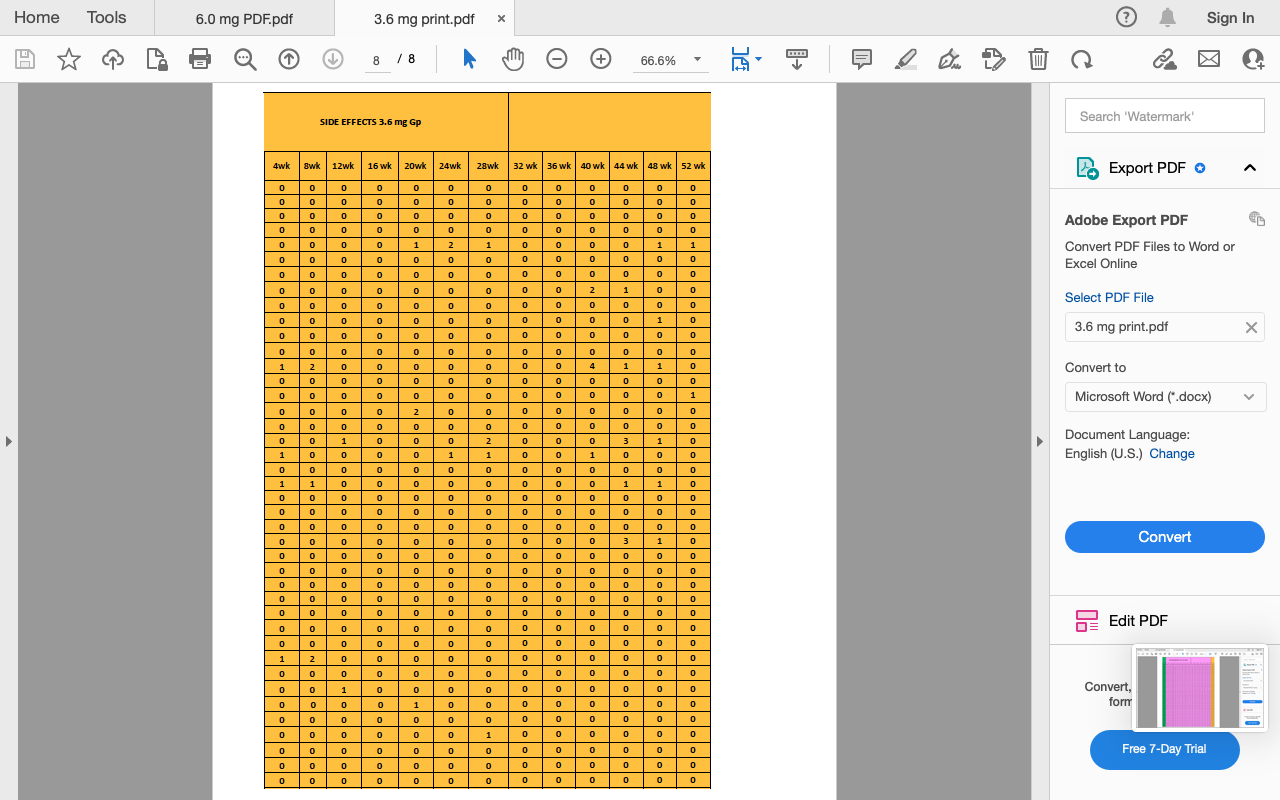


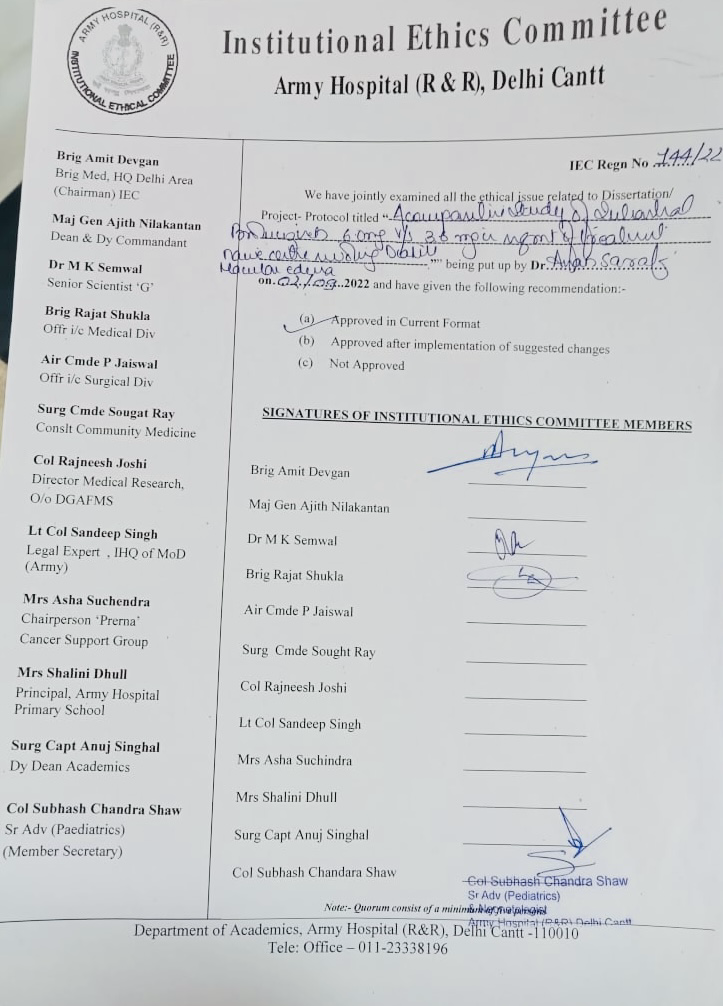
Appx B

Supplement: Supplementary file 1 — Supplementary Material 1. [file 40942_2025_628_MOESM1_ESM.zip › Supplements.docx]
